# Role of activins in embryo implantation and diagnosis of ectopic pregnancy: a review

**DOI:** 10.1186/1477-7827-12-116

**Published:** 2014-11-25

**Authors:** Bassem Refaat

**Affiliations:** Laboratory Medicine Department, Faculty of Applied Medical Sciences, Umm Al-Qura University, Al Abdeyah, Makkah, PO Box 7607, Saudi Arabia

**Keywords:** Activin, Fallopian tube, Endometrium, Implantation, Ectopic pregnancy, Early diagnosis

## Abstract

Embryo implantation is a major prerequisite for the successful establishment of pregnancy. Ectopic implantation outside the intrauterine cavity and the development of ectopic pregnancy (EP) is a major cause of maternal morbidity and occasionally mortality during the first trimester. EP may be induced by failure of tubal transport and/or increased tubal receptivity. Activins, their type II receptors and follistatin have been localised in the human endometrial and tubal epithelium and they are major regulators of endometrial and tubal physiology during the menstrual cycle. Pathological expression of activins and their binding protein, follistatin, was observed in tissue and serum samples collected from EP. Several studies with different designs investigated the diagnostic value of a single measurement of serum activin-A in the differentiation between normal intrauterine and failing early pregnancy and the results are controversial. Nevertheless, the diagnostic value of activins in EP, including the other activin isoforms (activin-B and –AB) and follistatin, merits further research. This review appraises the data to date researching the role of activins in the establishment of normal pregnancy and, pathogenesis and diagnosis of tubal EP.

## Background

The complex process of implantation and trophoblast invasion is crucial for the successful establishment of pregnancy [1–3]. Normally an embryo implants in the endometrium during the implantation window and uterine receptivity for implantation is maximal during this period [3]. Endometrial receptivity involves the expression of molecular factors that govern the feto-maternal dialogue and initiate decidualisation of the endometrial stroma [1, 2]. Candidate molecules, including cytokines and growth factors, are secreted by endometrial and tubal epithelial cells and have been described as major regulators of blastocyst implantation [4].

Ectopic pregnancy (EP) is a form of abnormal pregnancy in which the fertilised ovum implants outside the intrauterine cavity and the ampullary region of the fallopian tube is the most common site of implantation [5, 6]. EP represents 1-2% of all pregnancies in developed countries and haemorrhage from an EP due to tubal rupture remains the most common cause of maternal mortality in the first trimester of pregnancy in those countries [7–9].

Little is known about the reasons why embryos in some pregnancies implant within the fallopian tube whilst most implant eutopically within the endometrium of the uterus [4, 10]. Ectopic implantation has long been attributed to failure of the tubal transport mechanism. Either/or the rhythmic smooth muscle contraction and ciliary beat activity are believed to fail thereby leading to an EP. An alternative explanation for EP would be an increase in tubal epithelial receptivity as a result of delay in the tubal transport and impaired endometrial receptivity with ectopic implantation occurring following failure of the normal biological interactions between endometrium, fallopian tube and embryo [4, 10].

Quantitative measurement of human chorionic gonadotropin (hCG) in combination with transvaginal ultrasound is the currently accepted paradigm for clinical diagnosis and management of EP, with hCG monitoring being used to follow patients until complete resolution of the EP [7, 8, 11, 12]. EP can be treated surgically by laparotomy or laparoscopy, medically by methotrexate (MTX) injection and occasionally by observation alone [8]. MTX is the most cost effective and has similar effect as laparoscopic management on prospective fertility and recurrence of EP. However, it requires early detection of EP as it is only suitable in haemodynamically stable patients, with minimal or no symptoms, initial serum hCG ≤3000 IU/L, EP size <4 cm and absence of foetal cardiac activity [13–15].

It is believed that almost 50% of EP cases are initially misdiagnosed despite the use of high-resolution transvaginal sonography and sensitive assays for β-hCG [7, 8, 16, 17]. The delay in the diagnosis of EP eliminates the option of conservative management, could lead to tubal rupture, maternal morbidity and/or mortality. Hence, there is a compelling need to develop new markers and algorithms that provide a more sensitive and specific tool for the diagnosis of EP [11, 18, 19].

Activins and their binding protein, follistatin, are abundantly expressed in the female reproductive tract and they have recently been proposed as potential sensitive and specific markers for the diagnosis of ectopic pregnancy by several research groups [20–22]. The current review summarises the physiological actions of activins and follistatin in the endometrium, fallopian tube, embryo implantation and their clinical value in the diagnosis of normal and abnormal early pregnancies.

## Methods

‘Medline’ and ‘EMBASE’ were searched using the terms ‘endometrium’ , ‘fallopian tube’ , ‘implantation’ , ‘early pregnancy’ , ‘ectopic pregnancy’ , ‘tubal pregnancy’ , ‘early pregnancy loss’ , ‘failing early pregnancy’ , ‘receptivity’ , ‘decidualization’ , ‘implantation failure’ and ‘trophoblast’ in combination with ‘activin’ or ‘follistatin’ for studies published between 1987 and 2014. Publications in the past 6 years were mostly selected, but commonly referenced and important older publications were not excluded. The reference lists of articles identified by this search strategy were also searched and those judged as relevant were included. For a study to be included, it needed to be primarily focused on the expression of activins and their related molecules by the endometrial and tubal tissues, their roles in the establishment of normal and abnormal pregnancies and their serological diagnostic values in failing early pregnancies. Studies that were solely epidemiological in nature were excluded.

### Structure, cell signalling and regulation of activins bioactivities

Activins are members of the transforming growth factor-β family and they were originally isolated from porcine follicular fluid in 1986. They were termed after their stimulatory effect on the secretion of follicle stimulating hormone from the pituitary gland. Activins were later found to be secreted by all organs of the female reproductive system where they function as paracrine and autocrine factors to regulate a variety of reproductive functions [21, 23, 24].

**Figure 1:**
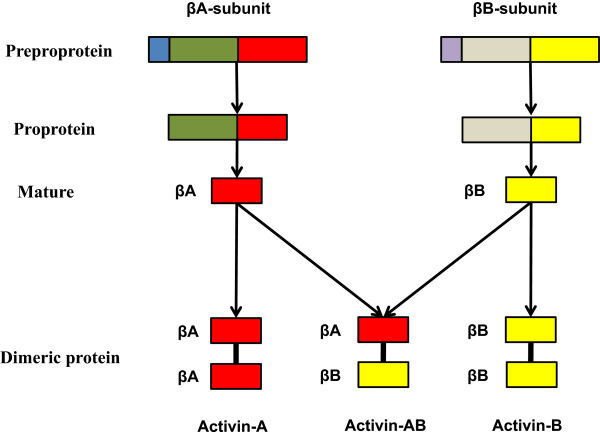
**Structure of activin mature dimer proteins.** The β-subunits are produced as larger precursor proteins, prepro-βA and -βB, that include a signal peptide and pro-region, both of which are cleaved to form the mature β-subunit. Activins are dimers consisting of two β-subunits joined by disulphide bridges.

Activins are homodimers or heterodimers of two β-subunits (βA and βB), and the different dimerization of subunits by a disulphide bond gives rise to three mature proteins named activin-A (βA-βA), activin-B (βB-βB) and activin-AB (βA-βB) (Figure 1) [23]. The human activin βA-subunit and βB-subunit genes are located on chromosome7 locus7p14-p15 and chromosome 2qcen-q13, respectively [25]. The mRNA of both subunits encodes a pre-proprotein, their mature regions illustrate about 70% sequence homology and both of them lack recognized glycosylation sites [26].

**Figure 2:**
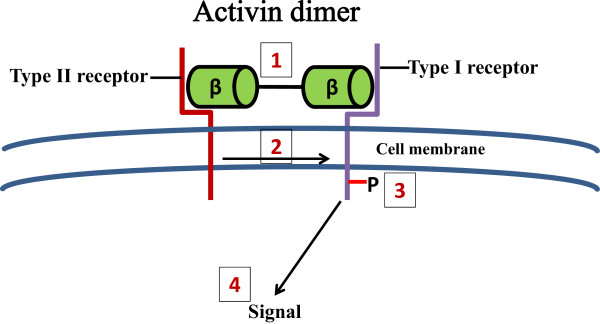
**The signalling mechanism of activin.** (1) Activin dimer binds to the activin type II receptors, (2) which in turn recruit and (3) phosphorylate the type I receptors. (4) The activin type I receptors transduce the activin signal to the nucleus.

Activins mediate their actions by binding to a complex of transmembrane serine and threonine kinase receptors. These activin receptors are classified into type I and type II receptor groups, comprising the activin type IIA and type IIB receptors (ActRIIA and ActRIIB) [23]. Activins can bind their individual receptor type II when expressed alone, but fail to bind to type I receptor in the absence of type II receptor [27]. However, both type I and II receptors are necessary to generate a high-affinity complex with the ligand, as well as for signalling (Figure 2) [28].

**Figure 3:**
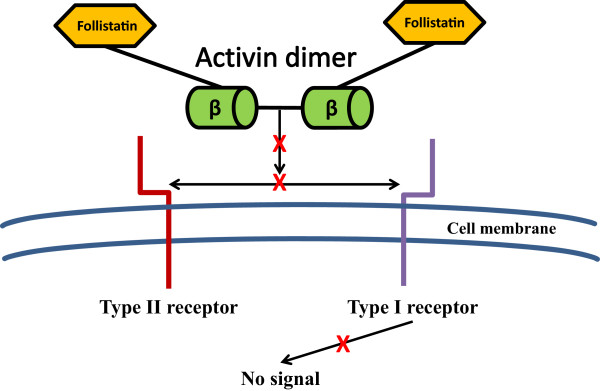
**The regulation of activin signalling by follistatin.** The activin-follistatin complex generally consists of one activin dimer and two follistatin molecules, which bind activin with high affinity and prevents binding of activin to its receptors.

The coordinated synthesis of follistatin with activin is the main regulator of the local bioactivity of activin since binding of activin to follistatin is almost irreversible [23]. The activin-follistatin complex generally consists of one activin dimer and two follistatin molecules [29]. In general, activin-A, −AB, and -B bind to follistatin with similar affinity (Figure 3) [30].

Both activin subunits, activin type II receptors and follistatin were previously localised within the human fallopian tube [23, 31], endometrium [32–34] and placental [35] tissues suggesting a role for these proteins in the regulation the physiological functions of these tissues. Therefore these proteins have been proposed as potential sensitive and specific markers to monitor the progress and outcome of pregnancy [36, 37].

### Roles of activins in normal early pregnancy events

Activins are potential factors for maternal–embryo interactions, due to their roles in regulating cell proliferation, differentiation and apoptosis, and their abundant expression and actions in remodelling tissues, embryogenesis and organogenesis in a variety of species [34, 38].

#### Activins in tubal physiology

Fertilisation and pre-implantation embryo development occur in the fallopian tube following ovulation and, the tubal epithelium is biologically active providing secretions to support these processes [23, 38]. The fertilised ovum travels through the fallopian tube under the control of tubal ciliary beat frequency and tubal smooth muscle contraction to reach to the intrauterine cavity for implantation within 48–72 h after fertilisation [39]. Impaired tubal transportation and secretory functions can occur after external or internal inflammation, leading to tubal damage, ectopic pregnancy and infertility [40].

Early reports on the expression of activin subunits in the fallopian tube were generated from different animal species. Originally, only βA-subunit expression was detected at the protein and gene levels in rat [41] and bovine [42] tubal tissues. Follistatin expression was not included in either study [41, 42]. Consistently, the expression of activins and follistatin by the human endosalpinx in pre- and postmenopausal women has been demonstrated [23, 31]. In all tissues studied, the βA- and βB-subunits were co-expressed with their type II receptors and binding protein, suggesting that the activin dimer has a local paracrine or autocrine role within the tube [23, 31].

Another study has later reported that the expression of both activin subunits mRNA and protein vary in a cycle-dependent manner in the oviduct of non-pregnant cycling mice [34]. The predominant source of activin β-subunits during the estrous cycle and pre-implantation phase were the epithelial cells lining the oviduct and endometrium [34]. A similar pattern of expression by the tubal epithelium during the menstrual cycle has also been reported in human fallopian tube and the expression of activin βA- and βB-subunits, activin type II receptors, and follistatin was highest in the luteal phase at the gene and protein levels. These results suggest that activins and their related molecules play a role in tubal physiology and early embryonic development [38].

The mouse and human oocyte is capable of receiving an activin signal produced by surrounding cumulus cells, but not of transmitting one [43]. This is further supported by the findings that early mouse and human embryos, from the four-cell stage to the morula stage, are unable to synthesise activins as no mRNA for these proteins has been identified at these stages of development [41, 44]. However, the expression of these molecules increased noticeably at the blastocyst stage, suggesting that the expression of the βA-subunit, activin type I receptor and follistatin is dependent on embryonic developmental stage and activation of the embryonic genome in mouse and human [44]. Therefore it has been postulated that activins, which bind to the activin type I and type II receptors on the oocyte and preimplantation embryo, may be derived from the surrounding tissue such as the tubal epithelium [34, 44].

At the present time, there is no evidence about a role for activins in the regulation of tubal ciliary beat frequency and smooth muscle contraction [6, 38]. However, several studies have shown that activin-A induces the production of nitric oxide, a potent inhibitor of tubal peristalsis, in a concentration-dependent manner in a variety of tissues and cells [45–47]. Furthermore, it has shown that activin-A decreased the expression of oxytocin and HoxA-10 mRNA by human myometrial cells in vitro and it decreased oxytocin and thromboxaneA2 induced accumulation of intracellular Ca^+2^ [48]. Therefore, activin-A could have a relaxing effect on human fallopian tube smooth muscle by stimulating the production of nitric oxide and/or by decreasing accumulation of intracellular Ca^+2^ [6].

#### Activins in endometrial physiology

The endometrium performs morphological and secretory adjustments during the menstrual cycle to form a receptive soil for the arriving blastocyst in the late luteal phase [1, 2, 4]. These alterations include the appearance of pinopodes in the epithelial layer, decidualization of the endometrial stroma and vascular proliferation, which are absolute requirements for successful implantation and establishment of communications with the embryo [1, 2, 4]. These modifications occur under the regulation of ovarian hormones to provide a microenvironment rich in cell adhesion molecules, cytokines, chemokines and growth factors [1, 4, 49, 50].

Activins have been described as important regulators of decidualisation and endometrial receptivity following the identification of their expression in the endometrium of several species at the gene and protein levels. Activin subunits are expressed in the endometrium luminal and glandular epithelium of several species including human [21, 34, 51, 52]. The co-expression of activin subunits, receptors and binding protein indicates that endometrial epithelial and stromal cells are capable of generating and responding to activin, and that there is a tight local regulation of activin actions within the endometrium [53].

The expression of activin and its related molecules varies during the menstrual cycle in the human endometrium as the uterus remodels and differentiates to form the decidua [54, 55]. Activin βA- and βB-subunits mRNA and protein are expressed by glandular and surface epithelium in non-pregnant endometrium [55] and, dimeric activin-A is present in uterine fluid [53] and menstrual blood [56] of cycling women. The expression of activin subunits is localised in the cytoplasm of endometrial epithelium and it significantly increases in the luminal and glandular epithelial cells during the secretory phase and remains relatively constant over the rest of the cycle [53, 55]. Furthermore, activin**-**A measured in endometrial washes collected from cycling women correlated significantly with menstrual cycle days and the thickness of endometrium [53].

A similar pattern of expression has also been observed in the stromal cells during the cycle and the expression was strongest in the late secretory phase where decidualisation and embryo implantation occur [54, 55]. Further evidence for the up-regulation of activin subunits synthesis with decidualisation was obtained from studies where the endometrium was extensively decidualised by intrauterine delivery of progestin [57] and in gene array studies examining decidualisation-related genes [58]. Newly decidualised cells are the main source of maternally derived activin-A during pregnancy to facilitate the spread of decidualisation throughout the endometrium by promoting the decidualisation of neighbouring cells [32–34, 57, 59].

During pregnancy, the placenta is a major source of serum activin-A, which increases as the pregnancy progresses [21, 24, 60, 61]. However, the expression of activins by the cytotrophoblast is low during early pregnancy suggesting that trophoblast invasion is induced by maternally derived activins [32]. Activin-A regulates trophoblastic cell adhesive properties by modulating the expression of E- and N-cadherin [62, 63] and integrins [35]. activin-A also promotes invasion of first-trimester cytotrophoblasts until 10 weeks gestation by increasing the expression of matrix metalloproteinases-2, 7 and 9 and its actions are inhibited by follistatin [33].

### Activins in the pathogenesis and diagnosis of EP

Studies on the expression pattern of activins, their type II receptors and follistatin by fallopian tubes bearing an ectopic pregnancy have shown a significant increase in βA-subunit, type IIA and IIB receptors and follistatin compared to normal control [6, 10]. Coherently, an increase in the expression of those molecules was also observed in tubal samples collected from patients with EP and who were positive for *Chlamydia trachomatis* antibodies when compared to tubal samples collected from EP patients and who had negative reaction for the antibodies [5]. Nevertheless, a recent study did not detect significant difference in the expression pattern of these molecules between implantation and remote sites collected from archived tubal pregnancy specimens. Hence, it has been postulated that the pathologic expression of activins and their related molecules by the tubal epithelium may play an important role in the pathogenesis of EP but not in the determination of implantation site [10].

Serum levels of activin-A and follistatin increase significantly throughout pregnancy and several studies have proposed a feto-placental origin for these proteins. Serum levels of activin-A also decrease in the presence of nonviable trophoblast [21, 64, 65]. Consequently, activin-A has been investigated as a potential marker for the diagnosis and differentiation between normal intrauterine pregnancy (IUP), miscarriage and ectopic pregnancy [37]. However, the reported results about the diagnostic value of activin-A are still debatable (Table 1).

**Table 1:** **Characteristics of published studies that investigated the diagnostic value of a single measurement of serum activin-A in the diagnosis of pregnancy of unknown location (PUL), ectopic pregnancy (EP), abortion (AB) and intrauterine pregnancy (IUP)**

Research group	Publication year	Study design	Number of patients	First trimester gestational age	Cut-off value	AUC	Sensitivity	Specificity	Reference number
**Florio et al.**	2007	Prospective Observational	536 PUL	Not reported	370 pg/mL	1.00	100%	99.6%	[36]
**Kirk et al.**	2009	Prospective Observational	141 PUL	Not reported	370 pg/mL	0.51	93%	13%	[71]
**Florio et al.**	2011	Retrospective case–control	30 EP & 30 IUP	Between 6 to 8 weeks	430 pg/mL	0.99	96.7%	100%	[66]
**Rausch et al.**	2011	Retrospective case–control	100 EP & 100 IUP	EP 45.15 ± 18.95; IUP 48.8 ± 18.34 days	376.15 pg/mL	0.78	80%	72%	[22]
**Warrick et al.**	2012	Retrospective cohort study	89 EP, 100 IUP & 100 AB	Median EP 5 weeks (1–10 weeks); AB and IUP 7 weeks (1–10 weeks)	< 260 pg/mL	0.62	59.6%	61%	[72]
**Roghaei et al.**	2012	Prospective case–control	100 EP & 100 IUP	EP 6.32 ± 1.03 weeks; IUP 6.85 ± 1.82 weeks	504 pg/mL	0.981	97%	93.5%	[69]
**Daponte et al.**	2013	Prospective case–control	30 EP, 30 AB & 33 IUP	Between 6 to 8 weeks	504 pg/mL	0.979	87.9%	100%	[67]

(AUC = area under the curve by receiver operator characteristics).

The initial study by Florio et al. (2007) [36] demonstrated that a single measurement of serum activin-A provided a highly sensitive and specific marker in 536 patients with pregnancy of unknown location (PUL) to discriminate between viable normal IUP, miscarriage and EP with a sensitivity of 100% and specificity of 99.6% at a cut-off value of 370 pg/mL. Later, the same research team has reported that serum concentrations of activin-A were significantly lower in 30 patients diagnosed with tubal EP when compared to control and, a sensitivity of 96.7% and a specificity of 100% for the diagnosis of EP was achieved at the cut-off level of 0.43 ng/mL [66]. These results have also been confirmed by other research groups in the following years but with a different cut-off value of 504 pg/mL that achieved a sensitivity of 97% and specificity of 93.5% [22, 67–70]. Additionally, Daponte et al. (2013) [67] also measured the sensitivity and specificity of follistatin in the diagnosis of EP. Their results have shown that both serum follistatin and activin-A/follistatin ratio had lower performance compared to serum activin-A in the diagnosis of EP.

Oppositely, another two studies reported that a single measurement of serum activin-A was neither sensitive nor specific for the diagnosis of early pregnancy failure including EP compared to β-hCG [71, 72]. Their results demonstrated that serum activin-A levels gave an area under the curve (AUC) of 0.61 for failing PUL, 0.64 for IUP and 0.51 for EP, and the model based on serum hCG levels gave an AUC of 0.95 for failing PUL, 0.97 for IUP and 0.67 for EP [71].

The inconsistencies in the reported cut-off values and the results of the different studies could be due to variability in trophoblastic activities and inadequate decidulisation during ectopic implantation [67]. It has also been suggested that some trophoblastic cells may behave as failing pregnancy during EP while other EPs could have more activity and behave more like IUPs [71], which could provide a possible explanation for the reported variations in serum levels of activin-A in women with EP [67].

Another explanation for the aforementioned variations in the results of activin-A could be related to the gestational age of the enrolled participants in the different studies. The increase in serum activin-A and follistatin during normal pregnancy starts at week 6 and reaches its peak at week 38 of pregnancy. The original study by Florio et al. (2007) [36]. included patients in the first trimester but the authors have not reported the gestational age for control, EP and miscarriage groups. In the following study by Rausch et al. (2011), the mean gestational age for EP and IUP was 45.15 ± 18.95 and 48.8 ± 18.34 days, respectively [22]. The most recent studies that have reported a diagnostic value for serum activin-A included only patients between 6 to 8 [67] and 5 to 6 weeks gestational age [70].

Alternatively, the two studies that showed no value for serum activin-A in the diagnosis of EP the gestational age for each group was not reported by Kirk et al. (2009) [71] and Warrick et al. (2012) [72] applied a retrospective design that included pregnant females with ≤10 weeks gestation. The ectopic pregnancy group in study reported by Warrick et al. (2012) [72] had a median of 5 weeks (range 1–10 weeks) gestation compared to 7 weeks (range 1–10 weeks) for both IUP and miscarriage. Therefore, future studies should categorise the patients with failing early pregnancy according to gestational age (e.g. ≤ and >6 weeks) and to compare the results with those obtained from gestational aged matched controls.

The biological activities of activins are tightly regulated by follistatin as the binding of activin to follistatin is almost irreversible [23]. Serum activin is commonly bound with the long form follistatin (FS-315) [29], while the short form (FS-288) has high affinity for cell membrane activins [73]. The currently available ELISA kits for the detection of activin-A and follistatin cannot distinguish between the free and bound forms of both proteins. Furthermore, the follistatin kit measures both the long and short forms. Therefore, the reported results from the different studies are shown at the level of total activin-A and follistatin and the development of ELISA kits that measure the free form of both proteins would expose precisely their values in the diagnosis of early pregnancy failure.

Additionally, the majority of studies that demonstrated the localisation of activins in the endometrium and placental tissues investigated the expression pattern of the βA- and βB-subunits and not the mature dimeric proteins. Hence, it could be postulated that the endometrium and trophoblast are cable of synthesising the different activin mature dimers (activin-A, −B and -AB). Moreover, generated results from gene knock-out studies have shown that each activin subunit has distinct functions and these subunits do not functionally overlap in all settings in vivo [74, 75]. Hence, the inclusion of the other activin mature dimeric proteins (e.g. activin-B and –AB) could add to the performance of serum activin-A in the diagnosis of abnormal early pregnancy.

## Conclusions

Ectopic pregnancy is worldwide health problem and it is the leading cause of maternal morbidity and/or mortality during the first trimester. Early diagnosis of EP would allow more conservative treatment approaches and preservation of prospective fertility. However, the currently used diagnostic modalities lack accuracy, reproducibility and simplicity in the prediction of EP.

Activins and their related proteins play an important role in the regulation of endometrial receptivity, trophoblast activity and embryo implantation. Pathological expression of these candidate proteins have been associated with abnormal implantation and failing early pregnancy at the tissue and serum levels. Although, the diagnostic value of a single measurement of serum activin-A in differentiating abnormal from normal pregnancy is controversial, it merits further research.

Future studies should classify the patients according to the gestational age and to include other activin mature dimer proteins. It could also be worthy to combine serum activins with other serum biomarkers (e.g. inhibins, vascular endothelial growth factor) for early and accurate diagnosis of EP. Additionally, the development of ELISA kits that measure the free form of activins could increase their diagnostic performances. Further studies are needed to develop biomarkers and diagnostic modalities for the early diagnosis of EP and prevention of its associated maternal complications.

## References

[1] Singh M, Chaudhry P, Asselin E (2011). Bridging endometrial receptivity and implantation: network of hormones, cytokines, and growth factors. J Endocrinol.

[2] Dimitriadis E, White CA, Jones RL, Salamonsen LA (2005). Cytokines, chemokines and growth factors in endometrium related to implantation. Hum Reprod Update.

[3] Coughlan C, Ledger W, Wang Q, Liu F, Demirol A, Gurgan T, Cutting R, Ong K, Sallam H, Li TC (2014). Recurrent implantation failure: definition and management. Reprod Biomed Online.

[4] Jia-Rong Z, Shuang-Di L, Xiao-Ping W (2009). Eutopic or ectopic pregnancy: a competition between signals derived from the endometrium and the fallopian tube for blastocyst implantation. Placenta.

[5] Refaat B, Al-Azemi M, Geary I, Eley A, Ledger W (2009). Role of activins and inducible nitric oxide in the pathogenesis of ectopic pregnancy in patients with or without Chlamydia trachomatis infection. Clin Vaccine Immunol.

[6] Refaat B, Amer S, Ola B, Chapman N, Ledger W (2008). The expression of activin-betaA- and -betaB-subunits, follistatin, and activin type II receptors in fallopian tubes bearing an ectopic pregnancy. J Clin Endocrinol Metab.

[7] Jurkovic D, Wilkinson H (2011). Diagnosis and management of ectopic pregnancy. BMJ.

[8] Sivalingam VN, Duncan WC, Kirk E, Shephard LA, Horne AW (2011). Diagnosis and management of ectopic pregnancy. J Fam Plann Reprod Health Care.

[9] Khan KS, Wojdyla D, Say L, Gulmezoglu AM, Van Look PF (2006). WHO analysis of causes of maternal death: a systematic review. Lancet.

[10] Refaat B, Simpson H, Britton E, Biswas J, Wells M, Aplin JD, Ledger W (2012). Why does the fallopian tube fail in ectopic pregnancy? The role of activins, inducible nitric oxide synthase, and MUC1 in ectopic implantation. Fertil Steril.

[11] Kao LY, Scheinfeld MH, Chernyak V, Rozenblit AM, Oh S, Dym RJ (2014). Beyond ultrasound: CT and MRI of ectopic pregnancy. AJR Am J Roentgenol.

[12] Kirk E, Bottomley C, Bourne T (2014). Diagnosing ectopic pregnancy and current concepts in the management of pregnancy of unknown location. Hum Reprod Update.

[13] Hajenius PJ, Mol F, Mol BW, Bossuyt PM, Ankum WM, van der Veen F (2007). Interventions for tubal ectopic pregnancy. Cochrane Database Syst Rev.

[14] Lipscomb GH (2007). Medical therapy for ectopic pregnancy. Semin Reprod Med.

[15] Bachman EA, Barnhart K (2012). Medical management of ectopic pregnancy: a comparison of regimens. Clin Obstet Gynecol.

[16] Dillon KE, Sioulas VD, Sammel MD, Chung K, Takacs P, Shaunik A, Barnhart KT (2012). How and when human chorionic gonadotropin curves in women with an ectopic pregnancy mimic other outcomes: differences by race and ethnicity. Fertil Steril.

[17] Morse CB, Sammel MD, Shaunik A, Allen-Taylor L, Oberfoell NL, Takacs P, Chung K, Barnhart KT (2012). Performance of human chorionic gonadotropin curves in women at risk for ectopic pregnancy: exceptions to the rules. Fertil Steril.

[18] Cartwright J, Duncan WC, Critchley HO, Horne AW (2009). Serum biomarkers of tubal ectopic pregnancy: current candidates and future possibilities. Reproduction.

[19] Cabar FR, Fettback PB, Pereira PP, Zugaib M (2008). Serum markers in the diagnosis of tubal pregnancy. Clinics (Sao Paulo).

[20] Barnhart K, Speicher DW (2011). Molecular diagnosis of ectopic pregnancy. Expert Rev Mol Diagn.

[21] Florio P, Luisi S, Ciarmela P, Severi FM, Bocchi C, Petraglia F (2004). Inhibins and activins in pregnancy. Mol Cell Endocrinol.

[22] Rausch ME, Sammel MD, Takacs P, Chung K, Shaunik A, Barnhart KT (2011). Development of a multiple marker test for ectopic pregnancy. Obstet Gynecol.

[23] Refaat BA, Bahathiq AO, Sockanathan S, Stewart RL, Wells M, Ledger WL (2004). Production and localization of activins and activin type IIA and IIB receptors by the human endosalpinx. Reproduction.

[24] Schneider-Kolsky M, D’Antona D, Evans LW, Taylor N, O’Connor A, Groome NP, de Kretser D, Wallace EM (2000). Maternal serum total activin A and follistatin in pregnancy and parturition. BJOG.

[25] Barton DE, Yang-Feng TL, Mason AJ, Seeburg PH, Francke U (1989). Mapping of genes for inhibin subunits alpha, beta A, and beta B on human and mouse chromosomes and studies of jsd mice. Genomics.

[26] Mason AJ, Hayflick JS, Ling N, Esch F, Ueno N, Ying SY, Guillemin R, Niall H, Seeburg PH (1985). Complementary DNA sequences of ovarian follicular fluid inhibin show precursor structure and homology with transforming growth factor-beta. Nature.

[27] Ebner R, Chen RH, Lawler S, Zioncheck T, Derynck R (1993). Determination of type I receptor specificity by the type II receptors for TGF-beta or activin. Science.

[28] Wrana JL, Tran H, Attisano L, Arora K, Childs SR, Massague J, O’Connor MB (1994). Two distinct transmembrane serine/threonine kinases from Drosophila melanogaster form an activin receptor complex. Mol Cell Biol.

[29] Schneyer AL, Hall HA, Lambert-Messerlian G, Wang QF, Sluss P, Crowley WF (1996). Follistatin-activin complexes in human serum and follicular fluid differ immunologically and biochemically. Endocrinology.

[30] Thompson TB, Lerch TF, Cook RW, Woodruff TK, Jardetzky TS (2005). The structure of the follistatin:activin complex reveals antagonism of both type I and type II receptor binding. Dev Cell.

[31] Bahathiq AO, Stewart RL, Wells M, Moore HD, Pacey AA, Ledger WL (2002). Production of activins by the human endosalpinx. J Clin Endocrinol Metab.

[32] Jones RL, Findlay JK, Farnworth PG, Robertson DM, Wallace E, Salamonsen LA (2006). Activin A and inhibin A differentially regulate human uterine matrix metalloproteinases: potential interactions during decidualization and trophoblast invasion. Endocrinology.

[33] Jones RL, Findlay JK, Salamonsen LA (2006). The role of activins during decidualisation of human endometrium. Aust N Z J Obstet Gynaecol.

[34] Jones RL, Kaitu’u-Lino TJ, Nie G, Sanchez-Partida LG, Findlay JK, Salamonsen LA (2006). Complex expression patterns support potential roles for maternally derived activins in the establishment of pregnancy in mouse. Reproduction.

[35] Stoikos CJ, Salamonsen LA, Hannan NJ, O’Connor AE, Rombauts L, Dimitriadis E (2010). Activin A regulates trophoblast cell adhesive properties: implications for implantation failure in women with endometriosis-associated infertility. Hum Reprod.

[36] Florio P, Severi FM, Bocchi C, Luisi S, Mazzini M, Danero S, Torricelli M, Petraglia F (2007). Single serum activin a testing to predict ectopic pregnancy. J Clin Endocrinol Metab.

[37] Prakash A, Laird S, Tuckerman E, Li TC, Ledger WL (2005). Inhibin A and activin A may be used to predict pregnancy outcome in women with recurrent miscarriage. Fertil Steril.

[38] Refaat B, Ledger W (2011). The expression of activins, their type II receptors and follistatin in human Fallopian tube during the menstrual cycle and in pseudo-pregnancy. Hum Reprod.

[39] Lyons RA, Saridogan E, Djahanbakhch O (2006). The effect of ovarian follicular fluid and peritoneal fluid on Fallopian tube ciliary beat frequency. Hum Reprod.

[40] Kriebs JM, Fahey JO (2006). Ectopic pregnancy. J Midwifery Womens Health.

[41] Lu RZ, Matsuyama S, Nishihara M, Takahashi M (1993). Developmental expression of activin/inhibin beta A, beta B, and alpha subunits, and activin receptor-IIB genes in preimplantation mouse embryos. Biol Reprod.

[42] Gandolfi F, Modina S, Brevini TA, Passoni L, Artini P, Petraglia F, Lauria A (1995). Activin beta A subunit is expressed in bovine oviduct. Mol Reprod Dev.

[43] Sidis Y, Fujiwara T, Leykin L, Isaacson K, Toth T, Schneyer AL (1998). Characterization of inhibin/activin subunit, activin receptor, and follistatin messenger ribonucleic acid in human and mouse oocytes: evidence for activin’s paracrine signaling from granulosa cells to oocytes. Biol Reprod.

[44] He ZY, Liu HC, Mele CA, Barmat L, Veeck LL, Davis O, Rosenwaks Z (1999). Expression of inhibin/activin subunits and their receptors and binding proteins in human preimplantation embryos. J Assist Reprod Genet.

[45] Nusing RM, Barsig J (1997). Inflammatory potency of activin A. Effect on prostanoid and nitric oxide formation. Adv Exp Med Biol.

[46] Lagadec P, Raynal S, Lieubeau B, Onier N, Arnould L, Saint-Giorgio V, Lawrence DA, Jeannin JF (1999). Evidence for control of nitric oxide synthesis by intracellular transforming growth factor-beta1 in tumor cells. Implications for tumor development. Am J Pathol.

[47] Nusing RM, Barsig J (1999). Induction of prostanoid, nitric oxide, and cytokine formation in rat bone marrow derived macrophages by activin A. Br J Pharmacol.

[48] Ciarmela P, Wiater E, Vale W (2008). Activin-A in myometrium: characterization of the actions on myometrial cells. Endocrinology.

[49] Horne AW, Critchley HO (2012). Mechanisms of disease: the endocrinology of ectopic pregnancy. Expert Rev Mol Med.

[50] Shaw JL, Horne AW (2012). The paracrinology of tubal ectopic pregnancy. Mol Cell Endocrinol.

[51] Sugawara K, Kizaki K, Herath CB, Hasegawa Y, Hashizume K (2010). Transforming growth factor beta family expression at the bovine feto-maternal interface. Reprod Biol Endocrinol.

[52] Zhang H, Nagaoka K, Imakawa K, Nambo Y, Watanabe G, Taya K, Weng Q (2013). Expression of inhibin/activin subunits in the equine uteri during the early pregnancy. Reprod Domest Anim.

[53] Florio P, Severi FM, Luisi S, Ciarmela P, Calonaci G, Cobellis L, Petraglia F (2003). Endometrial expression and secretion of activin A, but not follistatin, increase in the secretory phase of the menstrual cycle. J Soc Gynecol Investig.

[54] Mylonas I, Makovitzky J, Hoeing A, Richter DU, Vogl J, Schulze S, Jeschke U, Briese V, Friese K (2006). Inhibin/activin subunits beta-A (−betaA) and beta-B (−betaB) are differentially localised in normal, hyperplastic and malignant human endometrial tissue. Acta Histochem.

[55] Mylonas I, Jeschke U, Wiest I, Hoeing A, Vogl J, Shabani N, Kuhn C, Schulze S, Kupka MS, Friese K (2004). Inhibin/activin subunits alpha, beta-A and beta-B are differentially expressed in normal human endometrium throughout the menstrual cycle. Histochem Cell Biol.

[56] Reis FM, Nascimento LL, Tsigkou A, Ferreira MC, Luisi S, Petraglia F (2007). Activin A and follistatin in menstrual blood: low concentrations in women with dysfunctional uterine bleeding. Reprod Sci.

[57] Jones RL, Salamonsen LA, Zhao YC, Ethier JF, Drummond AE, Findlay JK (2002). Expression of activin receptors, follistatin and betaglycan by human endometrial stromal cells; consistent with a role for activins during decidualization. Mol Hum Reprod.

[58] Kao LC, Tulac S, Lobo S, Imani B, Yang JP, Germeyer A, Osteen K, Taylor RN, Lessey BA, Giudice LC (2002). Global gene profiling in human endometrium during the window of implantation. Endocrinology.

[59] Jones RL, Salamonsen LA, Findlay JK (2002). Activin A promotes human endometrial stromal cell decidualization in vitro. J Clin Endocrinol Metab.

[60] Fowler PA, Evans LW, Groome NP, Templeton A, Knight PG (1998). A longitudinal study of maternal serum inhibin-A, inhibin-B, activin-A, activin-AB, pro-alphaC and follistatin during pregnancy. Hum Reprod.

[61] O’Connor AE, McFarlane JR, Hayward S, Yohkaichiya T, Groome NP, de Kretser DM (1999). Serum activin A and follistatin concentrations during human pregnancy: a cross-sectional and longitudinal study. Hum Reprod.

[62] Ferreira MC, Witz CA, Hammes LS, Kirma N, Petraglia F, Schenken RS, Reis FM (2008). Activin A increases invasiveness of endometrial cells in an in vitro model of human peritoneum. Mol Hum Reprod.

[63] Li Y, Klausen C, Cheng JC, Zhu H, Leung PC (2014). Activin A, B and AB increase human trophoblast cell invasion by up-regulating N-cadherin. J Clin Endocrinol Metab.

[64] Luisi S, Florio P, D’Antona D, Severi FM, Sanseverino F, Danero S, Petraglia F (2003). Maternal serum inhibin A levels are a marker of a viable trophoblast in incomplete and complete miscarriage. Eur J Endocrinol.

[65] Muttukrishna S, Jauniaux E, Greenwold N, McGarrigle H, Jivraj S, Carter S, Elgaddal S, Groome N, Regan L (2002). Circulating levels of inhibin A, activin A and follistatin in missed and recurrent miscarriages. Hum Reprod.

[66] Florio P, Reis FM, Battista R, Luisi S, Moncini I, Bocchi C, Severi FM, Petraglia F (2011). Serum activin A levels are lower in tubal than intrauterine spontaneously conceived pregnancies. Gynecol Endocrinol.

[67] Daponte A, Deligeoroglou E, Garas A, Pournaras S, Hadjichristodoulou C, Messinis IE (2013). Activin A and follistatin as biomarkers for ectopic pregnancy and missed abortion. Dis Markers.

[68] Rausch ME, Barnhart KT (2012). Serum biomarkers for detecting ectopic pregnancy. Clin Obstet Gynecol.

[69] Roghaei MA, Sabet F, Mohamadi K (2012). Diagnostic accuracy of serum activin A in detection of ectopic pregnancy. J Res Med Sci.

[70] Elito Junior J, Gustavo Oliveira L, Octavio Fernandes Silva M, Araujo Junior E, Camano L (2014). Serum activin A levels and tubal ectopic pregnancy. Iran J Reprod Med.

[71] Kirk E, Papageorghiou AT, Van Calster B, Condous G, Cowans N, Van Huffel S, Timmerman D, Spencer K, Bourne T (2009). The use of serum inhibin A and activin A levels in predicting the outcome of ‘pregnancies of unknown location’. Hum Reprod.

[72] Warrick J, Gronowski A, Moffett C, Zhao Q, Bishop E, Woodworth A (2012). Serum activin A does not predict ectopic pregnancy as a single measurement test, alone or as part of a multi-marker panel including progesterone and hCG. Clin Chim Acta.

[73] Nakamura T, Sugino K, Titani K, Sugino H (1991). Follistatin, an activin-binding protein, associates with heparan sulfate chains of proteoglycans on follicular granulosa cells. J Biol Chem.

[74] Brown CW, Houston-Hawkins DE, Woodruff TK, Matzuk MM (2000). Insertion of Inhbb into the Inhba locus rescues the Inhba-null phenotype and reveals new activin functions. Nat Genet.

[75] Brown CW, Li L, Houston-Hawkins DE, Matzuk MM (2003). Activins are critical modulators of growth and survival. Mol Endocrinol.

